# Antigen-Capturing Mesoporous Silica Nanoparticles Enhance the Radiation-Induced Abscopal Effect in Murine Hepatocellular Carcinoma Hepa1-6 Models

**DOI:** 10.3390/pharmaceutics13111811

**Published:** 2021-10-29

**Authors:** Kyungmi Yang, Changhoon Choi, Hayeong Cho, Won-Gyun Ahn, Shin-Yeong Kim, Sung-Won Shin, Yeeun Kim, Taekyu Jang, Nohyun Lee, Hee Chul Park

**Affiliations:** 1 Department of Radiation Oncology, Samsung Medical Center, Seoul 06351, Korea; kyungmi.yang@samsung.com (K.Y.); chchoi93@gmail.com (C.C.); mementoamor@icloud.com (W.-G.A.); kkdnsy@naver.com (S.-Y.K.); camuserik@gmail.com (S.-W.S.); yeeun17.kim@sbri.co.kr (Y.K.); 2 School of Medicine, Sungkyunkwan University, Seoul 06351, Korea; 3 School of Advanced Materials Engineering, Kookmin University, Seoul 02707, Korea; gkdud5305@naver.com (H.C.); wkdxorb55@kookmin.ac.kr (T.J.)

**Keywords:** mesoporous silica nanoparticles, radiotherapy, immunotherapy, tumor microenvironment, abscopal effect

## Abstract

Immunomodulation by radiotherapy (RT) is an emerging strategy for improving cancer immunotherapy. Nanomaterials have been employed as innovative tools for cancer therapy. This study aimed to investigate whether mesoporous silica nanoparticles (MSNs) enhance RT-mediated local tumor control and the abscopal effect by stimulating anti-cancer immunity. Hepa1-6 murine hepatocellular carcinoma syngeneic models and immunophenotyping with flow cytometry were used to evaluate the immune responses. When mice harboring bilateral tumors received 8 Gy of X-rays on a single tumor, the direct injection of MSNs into irradiated tumors enhanced the growth inhibition of irradiated and unirradiated contralateral tumors. MSNs enhanced RT-induced tumor infiltration of cytotoxic T cells on both sides and suppressed RT-enhanced infiltration of regulatory T cells. The administration of MSNs pre-incubated with irradiated cell-conditioned medium enhanced the anti-tumor effect of anti-PD1 compared to the as-synthesized MSNs. Intracellular uptake of MSNs activated JAWS II dendritic cells (DCs), which were consistently observed in DCs in tumor-draining lymph nodes (TDLNs). Our findings suggest that MSNs may capture tumor antigens released after RT, which is followed by DC maturation in TDLNs and infiltration of cytotoxic T cells in tumors, thereby leading to systemic tumor regression. Our results suggest that MSNs can be applied as an adjuvant for in situ cancer vaccines with RT.

## 1. Introduction

In recent years, immunotherapy has gained interest as an option in cancer treatment. Immune checkpoint inhibitors targeting cytotoxic T lymphocyte-associated protein 4 (CTLA-4), programmed cell death protein 1 (PD1), and programmed cell death ligand 1 (PD-L1) have been approved by the United States Food and Drug Administration (FDA) and are considered promising systemic therapies based on clinical trials for various types of cancers [1]. Radiotherapy (RT) is one of the major types of cancer treatments and is known to modulate cancer immunity. Preclinical studies and some clinical cases have reported surprising results of the abscopal effect of combining immunotherapy with RT [2]. However, this has not yet become standard treatment in the clinic, and not all patients have experienced this positive outcome. Clinical trials to determine the optimal combination of RT and immunotherapy are ongoing.

Immunotherapy has been tested clinically for hepatocellular carcinoma (HCC), which is the second leading cause of cancer-related deaths worldwide [3,4,5]. Studies have shown positive results, with objective response rates of up to 20% [5]. Despite these encouraging results, immunotherapy does have clinical limitations, such as a relatively low response rate and acquired resistance [6]. In particular, immune checkpoint inhibitors are known to be less effective for HCC because of the lack of tumor-infiltrating lymphocytes and the immunosuppressive tumor microenvironment [7]. Thus, there is an unmet need to find innovative ways to enhance such treatment effects [8]. RT, one of the strategies to overcome this problem, has been considered as an option to boost the efficacy of immunotherapy for HCC [9]. Clinically, in a study investigating the role of RT in advanced HCC patients treated with nivolumab, patients with previous or concurrent RT showed better clinical outcomes than those without RT [10]. We previously studied a syngeneic murine HCC model and confirmed the abscopal effect and immunological mechanisms of the combination of RT and anti-PD1 antibody [11]. However, the mechanism by which RT stimulates the immune system is not yet clear, and effective immune modulation by RT itself needs to be tested to better understand the synergy between RT and immunotherapy. Researchers have suggested that RT induces immunogenic cell death and that this property could be useful for in situ vaccination [12].

Various types of engineered nanomaterials have been developed for medicinal purposes. Among them, mesoporous silica nanoparticles (MSNs) have attracted growing attention as nanocarriers for drug delivery or antigen targeting for vaccination. Owing to their large surface area and porous structure, MSNs possess the absorbing property of small biomaterials, such as proteins [13]. They effectively deliver absorbed biomaterials to target cells [14,15]. Researchers have made progress in the development of this property of MSNs for cancer treatment. In addition to chemotherapeutic drug delivery, MSNs have been studied for cancer vaccination, which could elicit an anti-cancer immune response by delivering cancer antigens to antigen-presenting cells [16].

In this study, we used a syngeneic murine HCC model to investigate anti-cancer immune stimulation by using MSNs in combination with RT. We tested the hypothesis that MSNs effectively deliver RT-releasing cancer antigens to local immune cells and investigated whether MSNs can enhance local tumor control and the abscopal effect.

## 2. Materials and Methods

### 2.1. Synthesis and Characterization of Nanoparticles

MSNs were synthesized via the sol-gel reaction of silane agents in the presence of structure-directing agents [17]. We dissolved 3 g of cetyltrimethylammonium chloride (CTAC, 25% solution, 12 mL, Sigma-Aldrich, St. Louis, MO, USA) and 60 mg of triethanolamine (Sigma-Aldrich, St. Louis, MO, USA) in 120 mL of deionized water. After heating to 95 °C, 2.25 mL of tetraethyl orthosilicate (TEOS, Acros Organics, Fair Lawn, NJ, USA) was added. After 2 h, the solution was cooled to room temperature, and the nanoparticles were collected by centrifugation at 11,000 rpm for 30 min and washed with ethanol three times. To extract residual structure-directing agents from the pores of MSNs, 1.3 mL of hydrochloric acid was added to the solution, which was refluxed for 3 h. For amine functionalization, 2.8 mL of (3-aminopropyl) triethoxysilane (APTES, 99%, Sigma-Aldrich, St. Louis, MO, USA) was added to the pore-extracted MSN solution and reacted for 3 h at 80 °C.

Fluorescently labeled MSNs were prepared by the co-addition of a fluorescence dye-conjugated silane agent with TEOS. The fluorescence dye-conjugated silane agent was synthesized by reacting 5 mg of fluorescein isothiocyanate (FITC, Sigma-Aldrich, St. Louis, MO, USA) with 44 µL of APTES in 1 mL of ethanol for 12 h in the dark. Immediately after the addition of TEOS (2.25 mL) to a solution containing CTAC (12 mL) and triethanolamine (60 mg), 250 µL of pre-conjugated FITC-APTES solution was added. After 2 h, the solution was cooled to room temperature, and the nanoparticles were collected by centrifugation at 11,000 rpm for 30 min and washed with ethanol three times. To extract residual structure-directing agents from the pores of MSNs, 1.3 mL of hydrochloric acid was added to the solution, which was refluxed for 3 h. For amine functionalization, 2.8 mL of APTES was added and reacted for 3 h at 80 °C.

The morphology of the MSNs was analyzed using a transmission electron microscope (JEM-2010, JEOL, Mitaka, Tokyo, Japan) at an accelerating voltage of 200 kV. A transmission electron microscopy (TEM) sample was prepared by dropping a diluted solution containing MSNs on a TEM grid, which was followed by air drying. TEM images were obtained without staining. The hydrodynamic sizes and zeta potentials of MSNs were measured by dynamic light scattering (Zetasizer ZS90, Malvern Instrument, Malvern, Worcestershire, UK). The Brunauer-Emmett-Teller (BET) surface area and pore volume of MSNs were measured by nitrogen (N_2_) adsorption using a BELSORP-mini II (BEL, Toyonaka, Osaka, Japan).

### 2.2. Measurement of Antigen-Capturing Capacity

Fluorescein-conjugated ovalbumin (F-OVA, Invitrogen, Carlsbad, CA, USA) was used as a model antigen to evaluate the antigen-capturing capacity of MSNs. MSNs (1 mg) were incubated with 0.2 mg of F-OVA at 4 °C for 24 h. Next, the mixture was centrifuged at 11,000 rpm for 15 min. The F-OVA content was measured using a spectrophotometer (RF-6000, Shimadzu, Nakagyo, Kyoto, Japan). The amount of adsorbed MSNs was calculated from the change in the amount of F-OVA before and after adsorption.

### 2.3. Cell Culture

Murine hepatoma Hepa1-6 cells were purchased from the American Type Culture Collection (ATCC, Manassas, VA, USA). Hepa1-6 cells were cultured in Dulbecco’s modified Eagle’s medium (DMEM, Gibco, Carlsbad, CA, USA) containing 10% fetal bovine serum (FBS, Gibco) at 37 °C under a humidified atmosphere of 5% CO_2_.

### 2.4. Measurement of Tumor Growth in Mice Co-Treated with MSNs and Radiation

Five-week-old male C57BL/6 mice were purchased from Orient Bio (Seongnam, Gyeonggi, Korea). All animal procedures were conducted in accordance with the appropriate regulatory standards under the study protocol (ID: 20181227001). To observe the abscopal effect, a bilateral syngeneic HCC model was established as previously described [11]. Briefly, Hepa1-6 cells (1 × 10^6^ cells) were injected into the right hind leg of the mice, and the same number of Hepa1-6 cells was injected into the left leg of the same mice 3 days after the first injection. The mice were randomized into four groups (*n* = 10 per group): (i) sham treatment, (ii) radiation treatment, (iii) MSN treatment and (iv) radiation + MSNs. The right leg bearing a Hepa1-6 tumor was given with sham treatment or irradiated with 8 Gy of X-ray 14 days after cell inoculation. Irradiation was performed using a linear accelerator (Varian Medical System, Palo Alto, CA, USA), as previously described [8]. The as-synthesized MSNs (400 μg per tumor) were intratumorally injected into the irradiated tumors twice on days 14 and 15 after cell inoculation. The size of the tumors in both legs was measured every 2–3 days using calipers. The tumor volume was calculated using the formula: volume (mm^3^) = (W^2^ × L)/2 (W, width (mm); L, length (mm)), as previously described [11,18]. The mice were sacrificed 42 days after cell inoculation.

### 2.5. Flow Cytometry Analysis

Tumors were harvested from both legs of the mice on days 20 and 42 after cell injection. A single-cell suspension was prepared as described previously [11]. Briefly, after the removal of red blood cells, the cells were fixed with Cytofix (BD554655, BD Biosciences, San Jose, CA, USA) for 30 min at 4 °C and resuspended in staining buffer (BD554656). The cells were permeabilized using the Fix/Perm kit (00-5523, eBioscience, San Diego, CA, USA). For T cell analysis, cells were stained with anti-Foxp3 (BD560408) and anti-IFNγ (BD557724) antibodies for 30 min at 4 °C. After washing, the cells were stained with antibodies specific for CD4 (BD552051), CD8 (BD560469), CD25 (BD551071), and CD45 (BD559864). Data were acquired using a BD FACSVerse flow cytometer (BD Biosciences) and analyzed using FlowJo software version 10.6.1 (Three Star Inc., Ashland, OR, USA).

For dendritic cell (DC) analysis, inguinal lymph nodes of the irradiated tumor side were harvested four days after irradiation, and DCs were isolated using the EasySep mouse plasmacytoid DC isolation kit (STEMCELL Technologies, Vancouver, BC, Canada). The DCs were stained with anti-CD11c (BD553801), anti-CD80 (BD560016), anti-CD86 (BD560582) and anti-MHC II (BD562363), and subjected to flow cytometry.

### 2.6. Pre-Incubation of MSNs with Irradiated Cell Conditioned Medium

Hepa1-6 cells were seeded in a 100 mm dish and irradiated with 100 Gy of γ-rays using an IBL437C blood irradiator (CIS Bio International, Gif-sur-Yvette, Essone, France). After 48 h of incubation without FBS, the conditioned medium (CM) was collected and centrifuged at 1500× *g* for 10 min to remove cell debris. The MSNs were resuspended at a concentration of 2.5 mg/mL in the cell culture supernatant and incubated for 72 h at 4 °C. After centrifugation at 11,000 rpm for 15 min, CM-incubated MSNs (CM-MSNs) were collected and washed twice with PBS. CM-MSNs were resuspended in PBS at a final concentration of 8 mg/mL.

### 2.7. Cellular Uptake of MSNs

The immortalized immature DC line JAWS II was purchased from ATCC. JAWS II cells were seeded onto a cover slip (Paul Marienfeld GmbH & Co. KG, Lauda-Königshofen, Baden-Württemberg, Germany) and incubated with 250 μg/mL of rhodamine-preloaded MSNs for two days. Cells were fixed with 4% formaldehyde and permeabilized with 0.01% Triton X-100. Cells were stained with Alexa-Fluor488-conjugated phalloidin (Life Technologies, Eugene, OR, USA) and DAPI (Sigma-Aldrich, St. Louis, MO, USA). Fluorescence images were acquired using a Zeiss Observer D1 fluorescence microscope (Carl Zeiss, Oberkochen, Baden-Württemberg, Germany). The activation of JAWS II cells by CM-MSNs was determined by flow cytometry. JAWS II cells (5 × 10^6^ cells) were seeded in 12-well plates and incubated with 100 or 200 μg/mL of CM-MSNs for two days. Cells were stained with anti-CD40 (BD562846), anti-CD80, anti-CD86, anti-MHC I (BD742859), anti-MHC II and anti-PD-L1 (12-5982-82, eBioscience, San Diego, CA, USA). Stained cells were analyzed by a BD FACSVerse flow cytometry.

### 2.8. Measurement of Tumor Growth in Mice Co-Treated with CM-MSNs and Anti-PD1

To test the immune-boosting effect of CM-MSNs, a single Hepa1-6 tumor model was established by injecting into the right hind leg of C57BL/6 mice. When the tumors were palpable, the mice were randomly divided into four groups (*n* = 4 per group): (i) MSNs + isotype IgG, (ii) MSNs + anti-PD1, (iii) CM-MSNs + isotype IgG and (iv) CM-MSNs + anti-PD1. Isotype IgG and anti-PD1 (BE0089 and BE0146; Bio X Cell, West Lebanon, NH, USA) were intraperitoneally administered at a dose of 2 mg/kg twice per week. MSNs or CM-MSNs (8 mg/kg) were subcutaneously injected into the left hind leg on the same day as the antibody injection. Tumor size was measured every 2–3 days using calipers, as described above. The mice were sacrificed 28 days after cell inoculation.

### 2.9. Immunohistochemistry

Formalin-fixed paraffin-embedded tumor tissue specimens were prepared as described previously [18]. For immunohistochemistry (IHC), the tumors were sliced into 4-μm-thick sections, deparaffinized in xylene, rehydrated in graded alcohol, and washed with 0.01 M PBS, pH 7.4. Heat-induced epitope retrieval was performed using citrate buffer (pH 6.0; Dako, Carpinteria, CA, USA), followed by blocking with a blocking buffer (Dako). The tissue sections were stained with anti-CD4 and CD8 antibodies, followed by incubation with horseradish peroxidase-conjugated secondary antibodies and 3,3′-diaminobenzidine substrate chromogen solution (DAB, Dako). IHC images were obtained using an Aperio ScanScope AT slide scanner (Leica Biosystems Inc., Buffalo Grove, IL, USA) and analyzed using ImageScope software 12.4.3 (Leica Biosystems, Buffalo Grove, IL, USA).

### 2.10. Statistical Analysis

All quantitative data are presented as the mean ± standard deviation (SD) or standard error of the mean (SEM). Statistical analyses were performed using Prism 9.2 (GraphPad Software, San Diego, CA, USA). *p*-values were calculated using one-way analysis of variance (ANOVA), Kruskal-Wallis test, or unpaired two-tailed *t*-test. Statistical significance was set at *p* < 0.05.

## 3. Results

### 3.1. Characterization of MSNs

**Figure 1:**
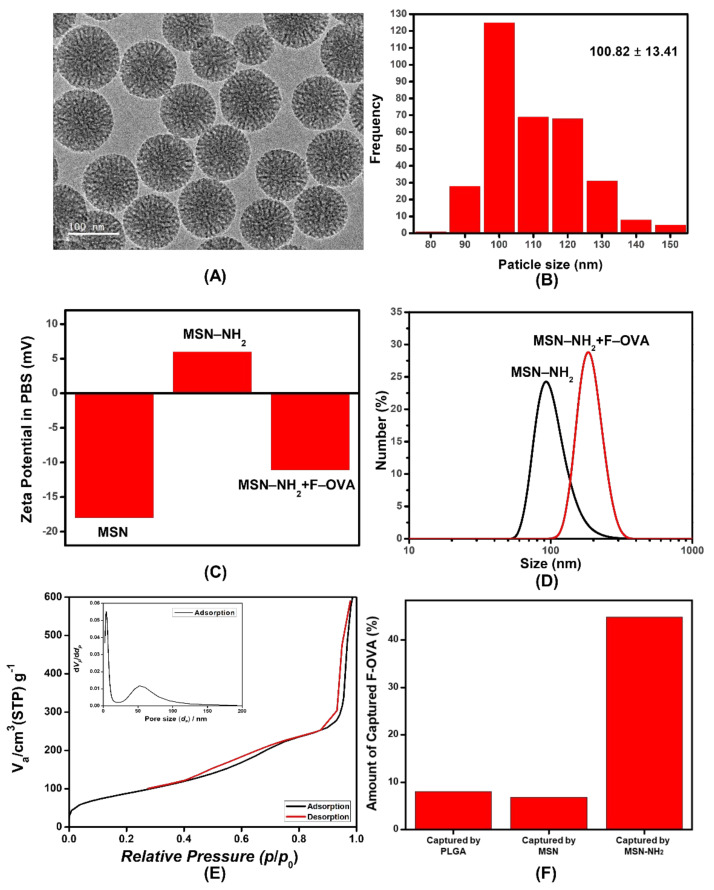
Synthesis of mesoporous silica nanoparticles (MSNs) with antigen-capturing capacity. (**A**) Transmission electron microscopy image of MSNs and (**B**) diagraph showing size distribution. (**C**) Zeta potentials of MSN, MSN-NH_2_ and MSN-NH_2_ after capturing fluorescein-conjugated ovalbumin (F-OVA). (**D**) Hydrodynamic diameter of MSN-NH_2_ before and after capturing F-OVA. (**E**) N_2_ absorption/desorption isotherms of MSNs. (Inset: pore size distribution from the adsorption branch). (**F**) Amount of F-OVA captured by poly-lactic-*co*-glycolic acid (PLGA), MSNs and MSN-NH_2_.

MSNs were synthesized via the sol–gel reaction of TEOS in the presence of CTAC as a pore-directing agent. TEM images showed that the MSNs had a spherical mesoporous structure (Figure 1A). The average size of MSNs was 100.82 ± 13.41 nm (Figure 1B). To optimize the antigen-capturing capacity, the surface of the MSNs was modified using APTES. The zeta potential of the strongly negative as-synthesized MSNs changed to a positive value after surface modification, confirming successful amine functionalization (Figure 1C). The hydrodynamic diameter of MSNs measured by dynamic light scattering was 104.22 nm (Figure 1D). The BET surface area and pore size determined via N_2_ adsorption/desorption experiments were 325.31 m^2^/g and 4.21 nm, respectively (Figure 1E). The antigen-capturing capacity of MSNs was estimated by the adsorption of F-OVA. Poly(lactic-*co*-glycolic acid) (PLGA) was employed as a control because its antigen-capturing capacity was recently reported [19]. After incubation of F-OVA with PLGA and MSNs, only 9% and 7% of F-OVA was adsorbed, respectively (Figure 1D). Surface modification with APTES significantly increased the affinity to F-OVA and 45% of F-OVA was captured by amine-functionalized MSNs. The interaction between MSNs and F-OVA can be attributed mainly to electrostatic interactions. After the adsorption of F-OVA, the zeta potential and hydrodynamic size of MSN were changed to −11.1 mV and 191 nm, respectively (Figure 1C,D). Owing to the enhanced capturing capacity, subsequent experiments were performed using amine-functionalized MSNs.

### 3.2. Effects of Intratumorally Injected MSNs on Radiation-Induced Abscopal Tumor Growth

**Figure 2:**
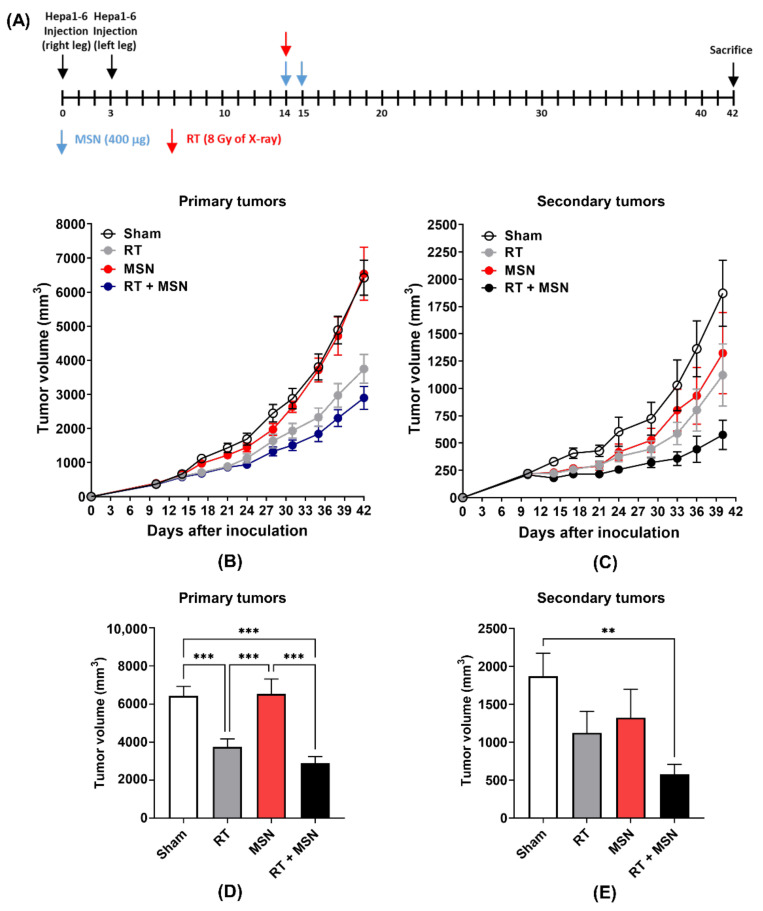
Intratumoral administration of MSNs enhances the radiation-induced abscopal effect in a bilateral Hepa1-6 tumor model. (**A**) Schedule of MSN or radiation treatment. (**B**) Growth curves of irradiated primary Hepa1-6 tumors implanted into C57BL/6 mice. (**C**) Growth curves of unirradiated secondary Hepa1-6 tumors in the same mice receiving RT. (**D**,**E**) Comparison of tumor volumes between treatment groups 42 days after tumor inoculation. ** *p* < 0.01; *** *p* < 0.001.

Given that MSNs with a tumor microenvironment can capture biomaterials released after RT, we tested the effect of intratumoral injection of MSNs on radiation-induced tumor growth inhibition. To observe the abscopal effect, the bilateral Hepa1-6 tumor model was used, as in a previous study [11]. Hepa1-6 cells were injected into both legs 3 days apart. X-rays (8 Gy) were delivered to the primary tumor site in the right hind leg, and MSNs were directly injected into the same primary tumor (Figure 2A). Radiation alone significantly decreased the growth of primary tumors but not secondary tumors (*p* < 0.001; Figure 2B–E). Intratumoral administration of MSNs did not affect the growth of unirradiated primary and secondary tumors, but it significantly decreased the growth of both primary and secondary tumors when injected into irradiated primary tumors (Figure 2B–E) compared with sham treatment. The difference in tumor volume between radiation and radiation plus MSNs was not statistically significant. These data indicate that MSNs in combination with RT inhibited the growth of distal and primary tumors.

### 3.3. Effects of Intratumorally Injected MSNs on Tumor-Infiltrating Lymphocytes

**Figure 3:**
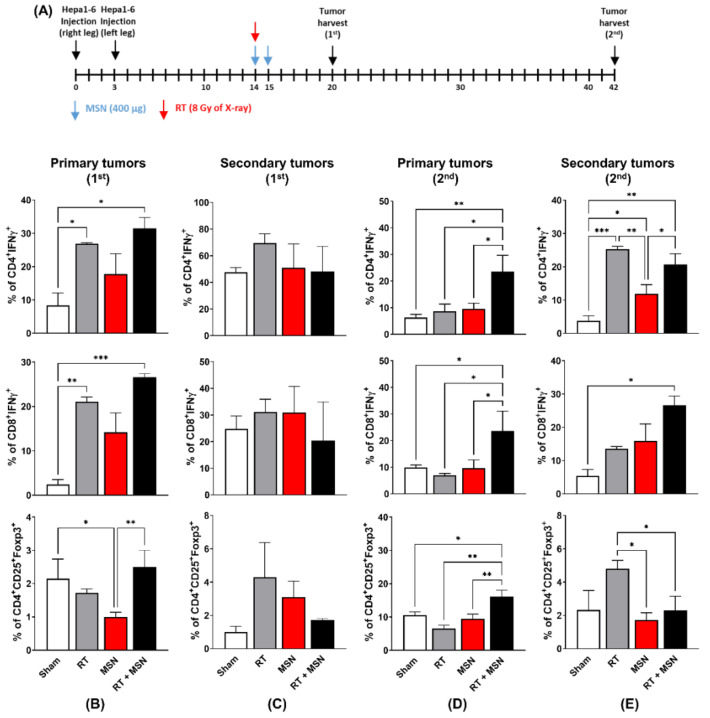
Direct injection of MSNs into irradiated primary tumors increases the number of cytotoxic T cells infiltrating the distal tumors. (**A**) Illustration of the treatment schedule and timing of tumor harvesting. (**B**,**C**) Profiling of activated T cells and Treg cells from primary (**B**) and secondary tumor tissues (**C**) harvested 20 days after tumor inoculation. (**D**,**E**) Profiling of activated T cells and Treg cells from primary (**D**) and secondary tumor tissues (**E**) harvested 42 days after tumor inoculation. * *p* < 0.05; ** *p* < 0.01; *** *p* < 0.001.

To further understand how MSNs facilitate radiation-induced abscopal effects, we evaluated the immunophenotype of tumor-infiltrating T lymphocytes. Primary and secondary tumors were harvested 6 and 28 days after irradiation (Figure 3A). Flow cytometric analysis revealed that in the tumors harvested on day 6, radiation increased the percentage of CD4+IFN*γ*+ T cells (*p* < 0.05) and CD8+IFN*γ*+ T cells (*p* < 0.05) in the primary tumors but not in the secondary tumors compared to the sham treatment (Figure 3B,C). Injection of MSNs into the irradiated primary tumors, but not the unirradiated tumors, further augmented the percentage of activated T cells, although this increase did not reach statistical significance. Instead, MSNs significantly decreased the number of CD4 + CD25 + Foxp3 + regulatory T cells (Tregs) in primary tumors (Figure 3B). In the tumors harvested on day 28, the number of activated CD4 + or CD8 + T cells significantly increased in the secondary tumors as well as the primary tumors co-treated with MSNs and radiation compared to that in sham or single treatments (Figure 3D,E). This correlates with a greater reduction in tumor size (Figure 2D,E). Radiation increased the number of Tregs in the unirradiated secondary tumors, which was suppressed by co-treatment with MSNs (Figure 3C,E). These data suggest that MSNs may enhance RT-induced tumor regression at the distal site via modulation of the T cell response.

### 3.4. Effects of MSNs on Dendritic Cell Activation

**Figure 4:**
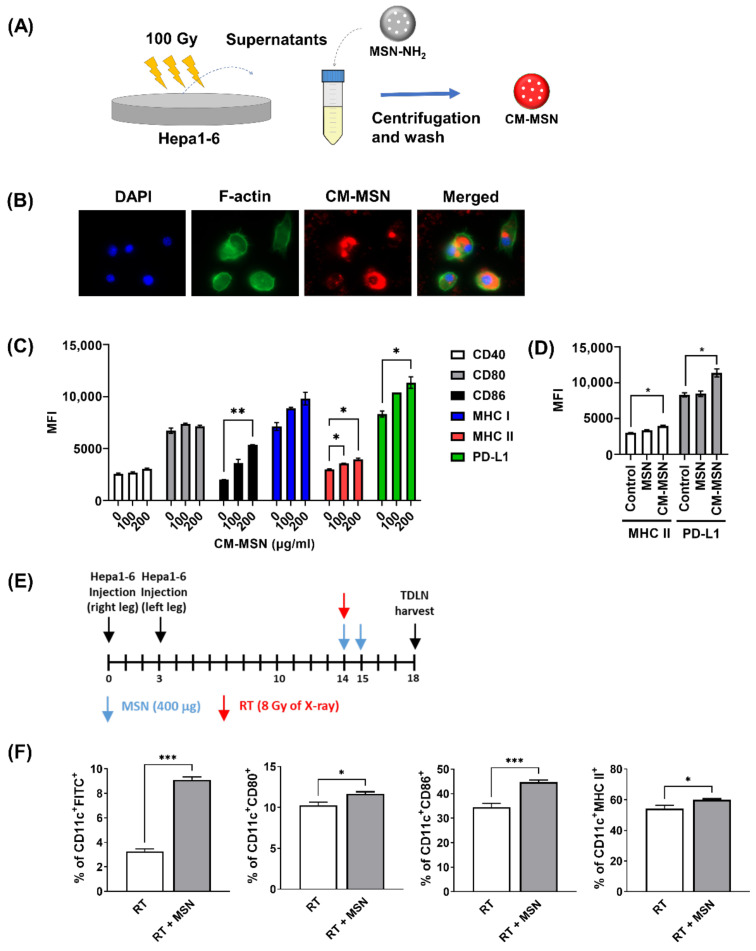
MSNs pre-incubated with the irradiated cell conditioned medium activates dendritic cells in vitro and in vivo. (**A**) Schematic diagram for the preparation of conditioned medium-incubated MSNs (CM-MSNs). (**B**) Uptake of CM-MSNs into JAWS II cells. Representative images of CM-MSNs labeled with rhodamine. (**C**) CM-MSNs activated JAWS II, an immortalized immature dendritic cell line. (**D**) MSNs without preconditioning did not activate JAWS II cells. (**E**) Schedule of timing of harvesting tumor-draining lymph nodes. (**F**) Flow cytometry data showing the MSN-increased population of activated DCs in TDLNs. * *p* < 0.05; ** *p* < 0.01; *** *p* < 0.001.

It is likely that the systemic anti-tumor effect of MSNs is linked to the activation of DCs residing in lymph nodes. To verify this, we tested whether MSNs could activate DCs in vitro. To recapitulate the in vivo situation, conditioned medium (CM) was prepared from Hepa1-6 cells irradiated with 100 Gy of γ-rays and incubated with MSNs to generate CM-MSNs (Figure 4A). First, to test whether DCs can take up CM-MSNs, immortalized immature dendritic JAWS II cells were incubated with rhodamine-preloaded CM-MSNs. Fluorescence imaging showed an efficient uptake of nanoparticles into the cytoplasm of JAWS II cells without visible toxicity (Figure 4B). Flow cytometry showed that CM-MSNs increased the surface expression of DC maturation markers, including CD86, MHC I and MHC II, in a dose-dependent manner (Figure 4C). CM-MSNs also upregulated PD-L1 expression in JAWS II cells. In contrast, MSNs without preconditioning in medium did not activate JAWS II cells at 200 μg/mL (Figure 4D).

Next, we examined whether MSNs injected into irradiated tumors facilitated DC maturation in tumor-draining lymph nodes (TDLNs) collected from mice. In mice bearing bilateral Hepa1-6 tumors, the right hind legs were exposed to X-rays (8 Gy) and then injected with FITC-preloaded MSNs or PBS. TDLNs were collected 3 days after irradiation and subjected to flow cytometry (Figure 4E). DCs obtained from mice co-treated with radiation, and MSNs showed an increase in the FITC-positive cell population compared to that in mice treated with radiation alone (*p* < 0.001; Figure 4F), suggesting uptake of MSNs by DCs. Injection of MSNs into irradiated tumors increased the expression of DC maturation markers such as CD80, CD86 and MHC II in DCs from TDLNs (Figure 4F), which is consistent with the in vitro results.

### 3.5. Effects of CM-MSNs on Hepa1-6 Tumor Growth in a Syngeneic Mouse Model

**Figure 5:**
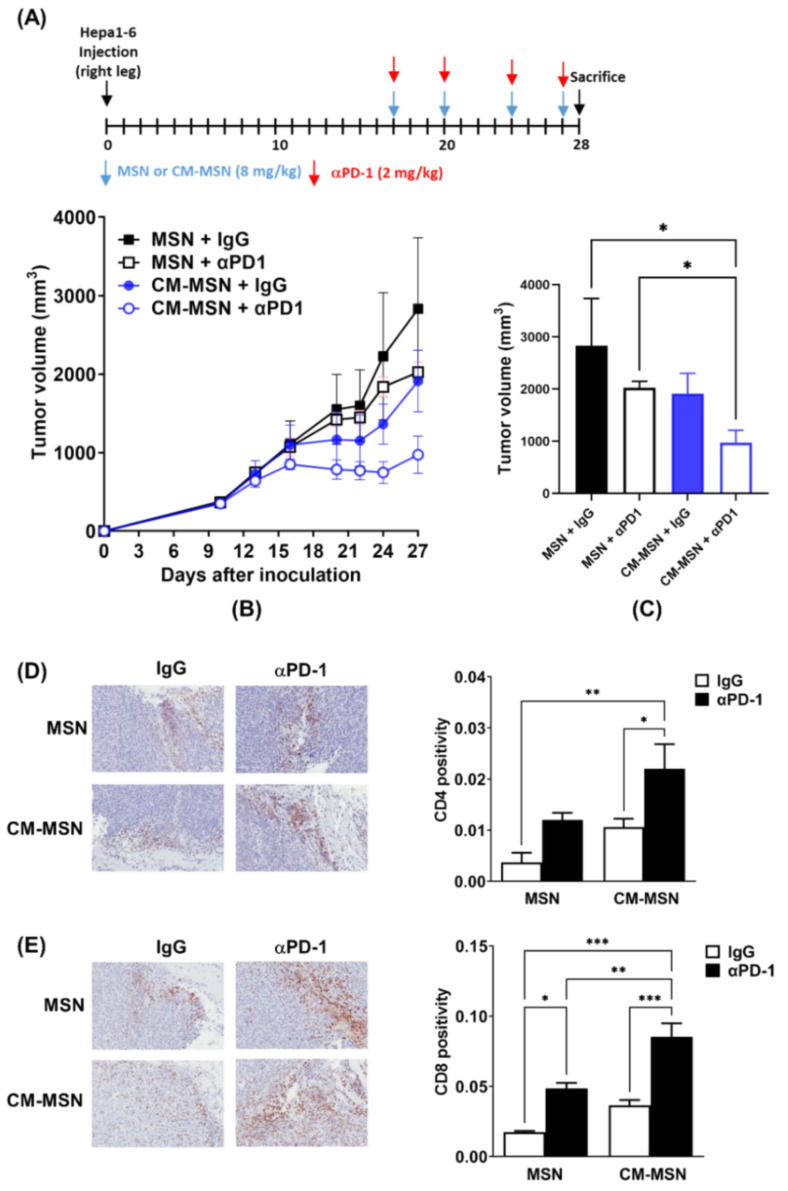
MSNs pre-incubated with irradiated cell conditioned medium augments the anti-tumor effect of programmed cell death protein 1 (anti-PD1) in a syngeneic hepatocellular carcinoma (HCC) model. (**A**) Schedule of MSN or anti-PD-1 treatment. (**B**) Growth curves of Hepa1-6 tumors implanted into C57BL/6 mice. (**C**) Comparison of tumor volume 27 days after tumor inoculation. (**D**) Representative immunohistochemistry (IHC) images and quantitative data of CD4 staining in Hepa1-6 tumor tissues. (**E**) Representative IHC images and quantitative data of CD8 staining in Hepa1-6 tumor tissues. * *p* < 0.05; ** *p* < 0.01; *** *p* < 0.001.

To test the function of CM-MSNs as a cancer vaccine therapy in vivo, we inoculated Hepa1-6 cells into the right hind legs and then evaluated the combined effect of the nanomaterials and anti-PD1 on tumor growth (Figure 5A). Treatment with CM-MSNs delayed tumor growth compared to treatment with the as-synthesized MSNs (Figure 5B,C). CM-MSNs augmented anti-PD1-mediated tumor growth inhibition to a greater extent than the as-synthesized MSNs (*p* < 0.05; Figure 5B,C). Immunohistochemical analysis of CD4 and CD8 in Hepa1-6 tumor tissues showed that CM-MSNs and anti-PD1 greatly increased the infiltration of both CD4- and CD8-positive cells within the tumor tissues (Figure 5D,E). These data suggest that CM-MSNs could be applied in cancer vaccine therapies to boost cancer immunotherapy.

## 4. Discussion

MSNs have a large surface area and porous structure that aid in absorbing nano-level materials such as drugs, proteins, and DNA fragments [15]. Based on this ability, numerous studies on MSNs have been conducted for various therapeutic purposes, including drug delivery, diagnosis and imaging [13,15]. MSNs have also been explored for cancer vaccination because MSNs that absorb tumor antigens increase the anti-tumor adaptive immune response [20]. Hong et al. [21] showed that the encapsulation of ovalbumin in MSNs enhanced DC internalization, lysosomal degradation escape, and cross-presentation, resulting in tumor growth inhibition. The efficacy and clearance of MSNs depend on their physical characteristics, such as surface charge, diameter, and pore size [13]. In terms of size, MSNs with diameters of 30–200 nm are efficient for drug loading, and those less than 100 nm are sufficient for lymph node drainage [21]. Larger pore sizes have shown better antigen-presenting functions [22]. In this study, we designed MSNs that can capture antigens released after RT and tested their efficacy in combination with RT. PLGA particles have been shown to improve the abscopal effect by capturing RT-releasing antigens [19]. The measurement of antigen-capturing capacity revealed that the as-synthesized MSNs adsorbed a similar amount of F-OVA as the PLGA particles. However, the amine functionalization of MSNs increased the absorption capacity by more than 6-fold. Since the interaction between MSNs and biomolecules depends on physicochemical properties such as electrostatic interactions, hydrogen bonding, and hydrophobic interactions, we expect that the antigen-capturing capacity can be further improved using various silane agents.

Based on the finding that MSNs efficiently captured F-OVA, we speculated that MSNs directly injected into tumors could absorb biomolecules from irradiated tumor cells, thereby eliciting anti-tumor immunity. In the bilateral Hepa1-6 tumor model, MSNs plus RT showed better regression of both primary and secondary tumors. Since MSNs alone did not show any significant effect on tumor growth, it is likely that the capturing process of RT-released antigens by MSNs is necessary for boosting systemic anti-tumor effects. CM-MSNs were prepared to mimic the MSN-absorbing antigens released during RT treatment in vivo. As expected, CM-MSNs showed better tumor control and boosted the anti-cancer effect of the anti-PD1 antibody compared with the as-synthesized MSNs. The action of CM-MSNs may be related to the increase in the number of CD4+ and CD8+ T lymphocytes infiltrating the tumor tissues, which was further enhanced by co-treatment with anti-PD1. These data suggest that MSNs could work as an effective nanocarrier for RT-released antigens and exert a systemic anti-tumor effect. Finally, we expect that CM-MSNs or a combination of MSNs and RT will make the outcome of immunotherapy much stronger and more sustainable.

The key steps in anti-cancer immunity are the maturation of DCs for antigen presentation and the activation of tumor-infiltrating T lymphocytes [23]. Similar to the mechanism of general vaccination, cell-mediated immune responses proceed as follows: drainage to lymph nodes, uptake by DCs, maturation of DCs, and presentation of peptide–MHC I complexes to CD8 + T cells, which are termed as the DUMP cascade process [24]. In this study, the uptake of CM-MSNs by DCs and subsequent DC activation in TDLNs was confirmed in vitro and in vivo (Figure 4). To test the early and late responses of T lymphocytes to RT and MSNs, tumors were harvested on days 6 and 26, and the immune cell populations were profiled (Figure 3). While the infiltration of cytotoxic T cells in the primary tumors was increased by either RT or RT plus MSNs in the early phase, there was no significant increased infiltration in secondary tumors. In the late phase, the number of cytotoxic T cells increased in the group treated with RT plus MSNs in both primary and secondary tumors, suggesting that MSNs may boost the RT-induced abscopal effect in the late phase. In addition, regulatory T cell numbers increased in the secondary tumors in the group that received RT alone, but not in the group treated with RT plus MSNs, which may endow effector T cells with cytotoxic activity. It could be inferred that MSNs balance immunity toward anti-tumor responses within the tumor microenvironment through continuous antigen delivery [14]. Taken together, our data suggest that MSNs capturing RT-induced antigens may strengthen the DUMP cascade, resulting in a significant suppression of both local and abscopal tumors via systemic anti-tumor immunity [21].

High doses of RT kill tumor cells, thus releasing large amounts of various antigens, including damaged double-stranded DNA, which is a powerful stimulator of the immune system [25]. The antigens that were released by RT and captured by MSNs may be non-selective. These antigens can be mixed with neoantigens from tumors and self-antigens shared with normal tissue [26,27]. However, theoretically, there might be no immune response to the materials from normal tissues as an immune tolerance [28]. This study found that CM-MSN or MSNs as adjuvants with RT acted in similar way as in vivo vaccines [29]. Despite many efforts, numerous typical cancer vaccines have failed [29]. One of the reasons is immune resistance to vaccine therapy because most cancer vaccines have a specific antigen target [30]. In contrast, in situ vaccines have the advantage of generating multiple antigens that can act simultaneously [31]. In addition, to overcome this resistance, various combination therapies, including immunotherapy, have been recently tested [29]. Our data on CM-MSNs might be consistent with the clinical data that hypofractionated RT or stereotactic body RT show better effects with immunotherapy [32]. Furthermore, MSNs may provide tumor antigens for longer periods [21], which is an important condition for successful therapeutic cancer vaccines.

The development of therapeutic nanoparticles has been successfully achieved through recent technical advances. However, clinical translation remains a challenge owing to several issues such as biocompatibility and safety. Fortunately, silica-based nanomaterials have been extensively tested because of their non-toxic nature [33]. After the injection of MSNs, their accumulation was found in the liver and spleen, and MSNs were mainly secreted by the intestine and kidneys [33]. Toxicity was rarely observed, although the properties of MSNs, such as their size, would be related to their safety [33,34]. The FDA approved the first human trial of multimodal silica nanoparticles termed “Cornell dots” in 2011 [35]. Thus, MSNs with novel multifunctionality have great potential to be tested in humans. Although biocompatibility needs to be tested, our study suggests that MSNs are promising for cancer treatment in combination with RT. However, questions still remain about optimal treatment settings, including optimal conditions of MSNs (pore size, injection dose, etc.) and combination with immunotherapy and RT (dose and timing) [15].

In this study, we confirmed the mechanism of stimulating the cancer immune system by MSNs with RT and showed their potential for combination with immunotherapy. Future studies will need to test MSNs combined with RT and immunotherapy.

## 5. Conclusions

In this study, we evaluated the combined effect of MSNs and RT on local tumor control and the abscopal effect using syngeneic murine HCC models. Our findings suggest that MSNs may capture tumor antigens released after RT, which is followed by DC maturation in TDLNs and the infiltration of cytotoxic T cells in tumors, thereby leading to systemic tumor regression. MSNs could be applied to in situ cancer vaccines with RT in the future.

## Data Availability

Not applicable.
